# Nitric oxide‑releasing porous titanium foams fabricated by scalable sintering–dissolution process for antibiofilm activity and cytocompatibility

**DOI:** 10.1038/s41598-026-47080-x

**Published:** 2026-04-22

**Authors:** Man Li, Pengcheng Zhu, Vahid Heravi Shargh, Jenny Aveyard, Manohar Koduri, Mark Hunter, Julia G. Behnsen, Yuyuan Zhao, Judith M. Curran, Raechelle A. D’Sa

**Affiliations:** 1 Department of Materials, Design and Manufacturing Engineering, University of Liverpool, Liverpool, L69 3GH UK; 2 School of Mechanical and Automotive Engineering, Ningbo University of Technology, Ningbo, 315211 China

**Keywords:** Sintering-dissolution process (SDP), Porous titanium (Ti) foams, Nitric oxide (NO), Antibiofilm activity, Antimicrobial, Orthopaedic implants, Biotechnology, Materials science, Microbiology

## Abstract

Orthopaedic implants require surfaces that resist bacterial colonisation while supporting host cell compatibility. In this study, we fabricated highly interconnected porous titanium (Ti) foams using a cost‑effective sintering–dissolution process (SDP) and functionalised them with 11‑aminoundecyltriethoxysilane (AUTES) and covalently tethered *N*-diazeniumdiolate nitric oxide (NO) donors. Structural characterisation by Scanning electron microscopy, X‑ray micro‑CT, and Brunauer–Emmett–Teller analyses confirmed a hierarchical porous architecture with ~ 73% total porosity and extensive internal surface area, enabling efficient chemical functionalisation. Chemiluminescence analysis demonstrated formulation‑dependent NO payloads and sustained release for over 15 h in bacterial culture medium. The NO‑releasing foams significantly reduced biofilm-associated *Escherichia coli* and *Staphylococcus aureus* compared with untreated controls, with the 20% AUTES/NO formulation showing the most persistent antibiofilm activity at 24 h (*p* < 0.05). Limited effects were observed against planktonic bacteria. Human mesenchymal stem cells (hMSCs) adhered to and remained viable on both unmodified and functionalised foams over 7 days, indicating cytocompatibility of the surface modification following NO release. These findings demonstrate that SDP-derived porous Ti foams can be functionalised for localised NO delivery and effective antibiofilm activity while maintaining initial hMSC compatibility, offering a scalable platform for multifunctional Ti-based implant surfaces.

**Supplementary Information:**

The online version contains supplementary material available at 10.1038/s41598-026-47080-x.

## Introduction

Orthopaedic disorders associated with trauma, infection, tumour resection, and age-related degeneration are increasing globally, creating a growing demand for durable, biologically integrated load-bearing implants. This trend is particularly evident in ageing populations: the median age in the European Union is projected to increase by 4.5 years between 2019 and 2050, with individuals over 65 rising from 90.5 to 129.8 million^1^. Consequently, between 3.1 and 2.4 million total hip and knee replacements are performed annually^2,3^. These projections underscore the need for next-generation implants that combine high strength, reduced modulus, long-term biocompatibility, infection resistance, and cost-effective manufacturability.

Titanium (Ti) and its alloys remain the clinical gold standard for load-bearing orthopaedics due to their favourable strength-to-weight ratio, corrosion resistance, and biocompatibility. However, conventional machining, forging, and casting typically produce dense, monolithic implants that are mechanically stiff and biologically inert. The mismatch between the Young’s modulus of solid Ti and natural bone leads to stress shielding and impaired load transfer^4^, contributing to poor osteointegration, aseptic loosening, and implant failure^5,6^. Approximately 20% of failures are infection-related, 18% result from poor integration, and over 10% require revision within 15 years^7^. Thus, despite excellent bulk properties, dense Ti implants often fail to achieve durable biological fixation and integration.

Porous metallic foams provide a promising solution by introducing controlled porosity and interconnectivity that better replicate the mechanical and biological microenvironment of bone. Reduced modulus, enhanced permeability, and increased surface area promote tissue ingrowth, vascularisation, and improved implant–bone coupling. In porous Ti specifically, interconnected pore networks enable mechanical interlocking^8,9^, while pore size and volume fraction can be tuned to match defect-specific mechanical requirements^10^. Increased surface area has also been shown to upregulate osteogenic markers *in vitro* and enhance biomechanical performance *in vivo*^11^. Importantly, scaffold architecture is not merely passive: hierarchical and anisotropic pores enhance stress transmission to adherent cells, generating local strains that stimulate osteogenic differentiation and accelerate regeneration^12^. Likewise, compliant, metamaterial-inspired foams with very low effective moduli further increase tissue strain and activate mechanosensitive pathways associated with osteogenesis and angiogenesis^13,14^. This represents a shift from inert structural supports towards mechanically instructive implants that actively guide repair.

Additive manufacturing (AM), including powder bed fusion and directed energy deposition techniques, enables precise control of pore geometry and patient-specific designs^15^. However, these approaches often require specialised equipment, high energy input, and substantial cost, limiting scalability. Powder-metallurgy space-holder methods offer a simpler and more economical alternative. The sintering–dissolution process (SDP), developed at the University of Liverpool^16^, enables fabrication of metallic foams with highly controllable porosity, pore size, and shape. Unlike melting-based approaches, SDP retains particulate features that increase surface roughness and specific surface area. Although widely applied in energy, aerospace, and civil engineering^17–20^, SDP remains underexplored for biomedical use. While mechanical properties were not evaluated in this study, previous reports on SDP-derived metallic foams demonstrate tunable modulus and compressive strength within ranges suitable for load-bearing applications^21,22^. In addition, the hierarchical surface topography of these structures may further promote protein adsorption, cell attachment, and tissue integration.

Despite improvements in mechanics and osseointegration, implant-associated infection (IAI) remains a major clinical challenge and a leading cause of revision surgery^23,24^. Following bacterial adhesion, biofilms form that resist immune clearance and limit antibiotic penetration^25^, often requiring up to 1000-fold higher antibiotic concentrations than planktonic cells^26^. Systemic antibiotic therapy risks toxicity and antimicrobial resistance, motivating localised, non-antibiotic strategies. Alternatives including chitosan, chlorhexidine, silver, and zinc have previously been immobilised on Ti surfaces for infection control^27–30^. Nitric oxide (NO), a natural immune mediator, exhibits broad-spectrum antimicrobial and antibiofilm activity and is a promising alternative to antibiotics^31–36^. However, due to its short half-life, controlled delivery of NO is challenging and necessitates use of donor molecules^37^.

Our previous work demonstrated that *N*-diazeniumdiolate NO donors can be tethered onto solid Ti using aminosilane linkers, providing antimicrobial efficacy against *Staphylococcus aureus* and *Pseudomonas aeruginosa* while supporting human foetal osteoblast growth after NO release^34^. Curran and coworkers have shown that long-chain aminosilanes containing pendant amine groups promote apatite formation and osteogenic differentiation of mesenchymal stem cells^38,39^. Based on these findings, we aim to tether the osteoinductive aminosilane 11-aminoundecyltriethoxysilane (AUTES)  onto porous Ti surfaces to increase osteogenic response and these N-terminated silanes can be coupled to an *N*-diazeniumdiolate NO donor for infection control.

An ideal orthopaedic scaffold should therefore integrate three complementary functions: mechanical compliance and interconnected porosity to support load transfer and tissue ingrowth, bioactive surface chemistry to promote osteogenesis, and localised antimicrobial activity to prevent infection. However, achieving uniform multifunctional coatings on complex three-dimensional porous architectures remains challenging.

Here, we evaluate SDP as a scalable manufacturing route for porous Ti foams and investigate whether these structures can be functionalised with an osseointegrative and antimicrobial coating. The foams were characterised by Scanning electron microscopy (SEM), X-ray micro-computed tomography (X-CT), and Brunauer–Emmett–Teller (BET) analysis, functionalised with AUTES and *N*-diazeniumdiolate NO donors; and evaluated for NO release, antimicrobial efficacy against planktonic and biofilm-associated *E. coli* and *S. aureus*, as well as cytocompatibility with human mesenchymal stem cells (hMSCs). These complementary analyses were selected to directly relate foam architecture (SEM/X-CT/BET) to functionalisation capacity (NO loading/release) and ultimately to biological performance.

## Materials and methods

### Materials

Commercially pure Ti powder with an average particle size of 45 µm was purchased from Ecka Granules Metal Powder Ltd (Wednesbury, UK). Food grade sodium chloride (NaCl) with a particle size range of 425–710 µm was purchased from E&E Ltd (Melbourne, Australia). 11-aminoundecyltriethoxysilane (AUTES, > 95%) was purchased from Fluorochem Ltd (Hadfield, UK). All common laboratory solvents and salts, including ethanol, acetic acid (HOAc), nitric acid (HNO_3_), hydrochloric acid (HCl), disodium phosphate and sodium acetate (NaAc), with analytic grade were from Merck KGaA (Darmstadt, Germany) and used as received.

### Manufacturing of Ti foams

**Fig. 1:**
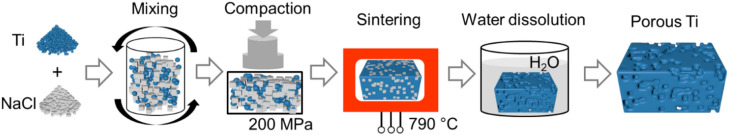
Schematic of the SDP process for manufacturing Ti foams.

Ti foams were prepared using the SDP process as previously described by Zhao et al.^16^. Briefly, Ti and NaCl powders were mixed at a volume ratio of 4:6 to manufacture porous samples with a porosity of 73%. The mixture was then compacted into preforms in cylindrical steel moulds using a hydraulic press at a pressure of 200 MPa. The preforms, together with the moulds, were sintered in an electric furnace at 790 °C for 4 h, followed by cooling in the air to room temperature. The sintered specimens were removed from the moulds and placed in a warm, running water stream for ca. 45 min to dissolve the NaCl particles embedded in the Ti matrices. A large porous Ti foam was obtained after removing all the NaCl particles by the dissolution route in water. The as-produced Ti foam was cut into disc shapes, with a diameter of 6 mm and a height of 2 mm using an electrical discharge machine Prima E250 (ONA Ltd., Bristol, UK). The Ti foams were washed in HNO_3_ and deionised water, then fully dried after autoclave sterilisation and stored in a desiccator before use. The SDP process is illustrated in Fig. 1.

### Silanisation and *N*-diazeniumdiolate-functionalisation

Cleaned Ti foams were immersed in different concentrations of (1, 5, 10, and 20% v/v) AUTES/ethanol solutions and slowly vacuumed where no bubble appeared in solution and subsequently shaken on a gyro-rocker (SSL3, Stuart) at 70 rpm for 4 h. The samples were then washed with ethanol for 3 × 5 min to remove unreacted silane and cured in an oven at 80 °C for another 4 h.

Silanised foams were functionalised with the NO-donor *N*-diazeniumdiolate in an in-house built stainless-steel NO reactor as previously reported^21^. Briefly, the reactor chamber was purged with 6 bars of argon (BOC, Guildford, UK) for 5 min (3x) and 10 min (3x) to remove atmospheric oxygen and water. Then NO (BOC, Guildford, UK) was introduced into the reactor at 5 bar for 3 days. At the end of this time, the residual NO was removed by flushing the chamber with 6 bar (0.6 MPa) argon for 5 min (2x) and 10 min (2x).* N*-diazeniumdiolate-functionalised samples were stored at − 20 °C until used.

Sample nomenclature is as follows: (a) the clean Ti foam termed as m_Ti; (b) silanised m_Ti termed using the concentration of AUTES in silanisation, for example m_Ti silanised in 1% concentration of AUTES termed as 1%AUTES; (c) *N*-diazeniumdiolate-functionalised samples are termed as m_Ti/NO, 1%AUTES/NO, 5%AUTES/NO, 10%AUTES/NO and 20%AUTES/NO. It should be highlighted here that there is no possible amine functionality on m_Ti which was used as a control group in the work, and the nomenclature of m_Ti/NO is used for consistency and readability.

### Scanning electron microscopy (SEM)

The morphology of the Ti foam was examined using SEM. m_Ti was coated with gold using a Q150T ES sputter coater, and images were captured on a JSM 7001F field-emission SEM with a voltage of 10.0 kV. The energy dispersive X-ray spectroscopy (EDX) was also used to characterise the elemental composition of m_Ti.

### X-ray computed tomography (X-CT) and structural analysis

X-ray CT scans of m_Ti foams were acquired using a Zeiss Xradia Versa 620 X-ray microscope. A large field-of-view scan was performed on an entire m_Ti foam, using the 0.4X objective, with a voxel size of 8.7 µm (detector binning 2). A source accelerating voltage of 80 kV at 10 W was used and an LE4 beam filter was applied. The scan comprised 1601 projection images with an exposure time of 1 s per image. Subsequently, a second higher-resolution scan of the central region of the m_Ti foam was performed with the 4X objective, achieving a voxel size of 2.35 µm. This scan was performed with a source voltage of 120 kV at 17.5 W, LE4 filter, and comprised 2401 projection images of 1 s exposure time each. The projection data were reconstructed in 3D using Zeiss Scout-and-Scan Reconstructor 16.1. Porosity and thickness analyses were performed using Fiji^40^, utilising both the BoneJ^41^ and MorphoLibJ^42^ plugins. A cubical region of interest was cropped from both datasets and the pores were segmented. Overall porosity was measured as the volume fraction (BoneJ) and Ti foam strut thickness with BoneJ’s Thickness option. Individual pore sizes were measured by separating the pores with a distance-map based watershed algorithm from MorphoLibJ and calculating the equivalent spherical diameter of the volume of each pore. 3D visualisation images were generated with Drishti^43^.

### Brunauer–Emmett–Teller (BET) analysis

The surface area of Ti foams was determined using a 3Flex 3500 gas sorption analyser at − 196 °C^18^. m_Ti and the silanised foams were degassed at 300 °C under vacuum for 3 h before measurement. Measurements of nitrogen adsorption were taken across the relative pressure (P/P0) range of 0 to 1, with measurements in the relative pressure range of 0 to 0.3 being used in BET analysis. Each sample was measured in triplicate.

### Chemiluminescence

The NO release from the Ti foams was measured using a Sievers 280i Chemiluminescence NO Analyzer (NOA280i, GE, USA). The *N*-diazeniumdiolate-functionalised samples (6 mm diameter, 2 mm thickness) were immersed in 5 mL of acetate buffer (pH 4) or LB broth bacterial culture medium (pH 7) at room temperature in a three-neck round bottom flask. Nitrogen gas was continuously sparged through the buffer at a flow rate of 150 mL/min. A vacuum pump connected to the NOA was used to draw the mixed gases into the reaction cell and maintain its pressure of the reaction cell. NO release was measured at intervals of 1 s for > 20 h.

### Antimicrobial activity assay on planktonic bacteria and biofilm eradication assay

*Escherichia coli* ATCC 10536 and *S. aureus* ATCC 25923 were used to evaluate the antibacterial efficiencies of the NO-releasing foams. The overnight cultures of *E. coli* and *S. aureus* were diluted to 1 × 10^6^ CFU/mL in LB broth, according to the absorbance (λ = 600 nm) and a 0.5 McFarland Standard^44^. Ti foams were placed in a 24-well plate and 1 mL of diluted bacterial solution was added to each well before incubation at 37 °C to allow bacterial growth. After 4 h and 24 h of incubation, the liquids in the well plates were transferred to another aseptic well plate and serially diluted with LB broth. For biofilm growth inhibition, foams were vigorously washed once with PBS to remove any unattached planktonic bacteria, and then 1 mL of fresh LB broth was used to remove and re-suspend the bio-film-associated bacteria after 4 h and 24 h incubation. Antimicrobial activity was determined using the Miles and Misra method on LB agar plates^45^. Serial dilutions of bacterial solution from planktonic or biofilm assay were collected, 3 × 10 µL of each dilution were dropped onto LB agar plate. Plates were moved out after overnight incubation, counting under room light for colonies grown on the agar plate. Experiment has been performed with three technical (n) and biological (N) replicates.

### Immunofluorescence live/dead assay

Bacterial cell viability was determined using the Live/Dead assay kit (L7007, ThermoFisher). After 4 h of incubation with bacteria, Ti foams were transferred to a new aseptic well plate and rinsed with sterile PBS to remove unattached bacteria. Next, 300 µL of combined reagent solution comprising of 5 µM SYTO 9 dye and 3 µM Propidium iodide was added to each well. The cells were then incubated in the dark at room temperature for 30 min. Finally, images were captured using a confocal laser-scanning microscope (LSM 510, Zeiss, Germany) with excitation/emission at 480/500 nm for SYTO9 stain and 490/635 nm for Propidium iodide. Three images were taken per sample and processed using ImageJ 1.48 software.

### hMSCs culture, subculture and seeding

hMSCs were purchased from Lonza Systems and cultured in growth medium consisting of DMEM with low glucose, supplemented with 10% FBS (Gibco), 2% penicillin–streptomycin (AppliChem), and 1% L-glutamine (200 mM solution). Cultured cells at > 90% confluency were trypsinised, counted and diluted in DMEM to give a cell seeding density of 30,000 cells/per test scaffold. All cultures were maintained at 5% CO_2_, 95% air, and 37 °C.

### Calcein AM and MTT cell viability assay

hMSCs that were cultured in contact with the test substrates were exposed to (3-(4,5-dimethylthiazol-2-yl)-2,5-diphenyltetrazolium bromide) (MTT) (1 mg/mL) and incubated at 37 °C, 5% CO_2_ (humidified atmosphere) for 4 h. The culture medium was then replaced with a solubilisation solution of DMSO (1 mL) and mixed on gyro rotor to dissolve the formazan crystals at room temperature for 20 min and the optical density was measured at 570 and 630 nm reference on a plate reader, with three technical and biological replicates (n = 3). Blank Ti foams with 30,000 cells/well were used to normalise the viability.

A 1 μL of Calcein AM stock solution (1 μg/1 μL) was dissolved in 1 mL of FBS-free medium to create a working solution. 1 mL of the working solution was added to the test sample and cultured for 30 min at 37 °C. The working solution was removed from the test samples, which were then washed with PBS prior to visualisation using confocal microscopy (LSM 510 Carl Zeiss) with an excitation wavelength of 488 nm and an emission max of 520 nm. Three images were taken per sample and each sample was carried out in triplicate and representative images are shown.

### Statistical analysis

One-way analysis of variance (ANOVA) was used to analyse the differences among the various treatment samples. The Student–Newman–Keuls (SNK) method was used to determine the significance between treatment types. All data were collected in triplicate and are presented as the mean ± standard deviation. Differences were considered statistically significant at *P* < 0.05.

## Results

### Characterisation of Ti foams

#### Morphology and elemental analysis of Ti foams

**Fig. 2:**
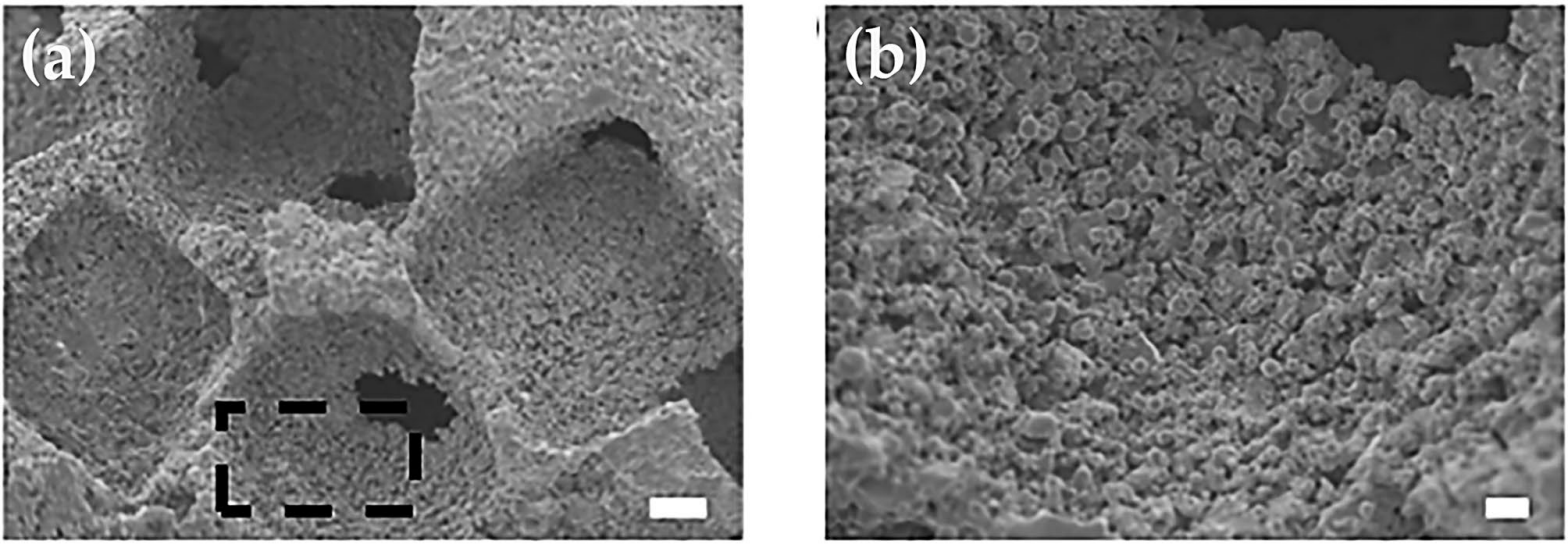
(**a**) SEM image of SDP m_Ti (scale bar = 100 µm) and (**b**) magnified view of the rectangular dashed region (scale bar = 20 µm).

The morphology and pore architecture of the SDP-derived Ti foams were first examined by scanning electron microscopy (SEM) (Fig. 2), with elemental composition assessed by EDX spectroscopy (Supplementary Fig. S1). SEM images revealed a homogeneous distribution of macropores with diameters of approximately 500 µm throughout the m_Ti foams. Smaller interconnecting pores (~ 100–200 µm) were also observed linking the larger voids (Fig. 2a), producing a continuous and highly interconnected network that is favourable for tissue ingrowth and vascularisation^45,46^. At higher magnification (Fig. 2b), the pore walls exhibited a roughened surface morphology characteristic of sintered Ti particles covered by a native oxide layer. This micro- and nanoscale roughness is expected to increase surface area and promote protein adsorption and cell attachment and also provides additional reactive sites for subsequent silane grafting and *N*-diazeniumdiolate tethering . Overall, the structure displayed hierarchical porosity, with pore sizes ranging from approximately 100 to 500 µm, consistent with metallic foams designed to mimic cancellous bone and support cell adhesion, proliferation, and differentiation^47–49^.

EDX analysis confirmed the chemical composition of the m_Ti foams (Supplementary Fig. S1). Only titanium and oxygen were detected, indicating complete removal of NaCl following dissolution. Oxygen and Ti were present at 15.7% and 84.3% by weight, and 35.8% and 64.2% by atomic percentage, respectively. The Ti/O atomic ratio of 1.8 is consistent with formation of a native surface oxide during processing.

### X-ray CT 3D imaging and structural analysis

**Fig. 3:**
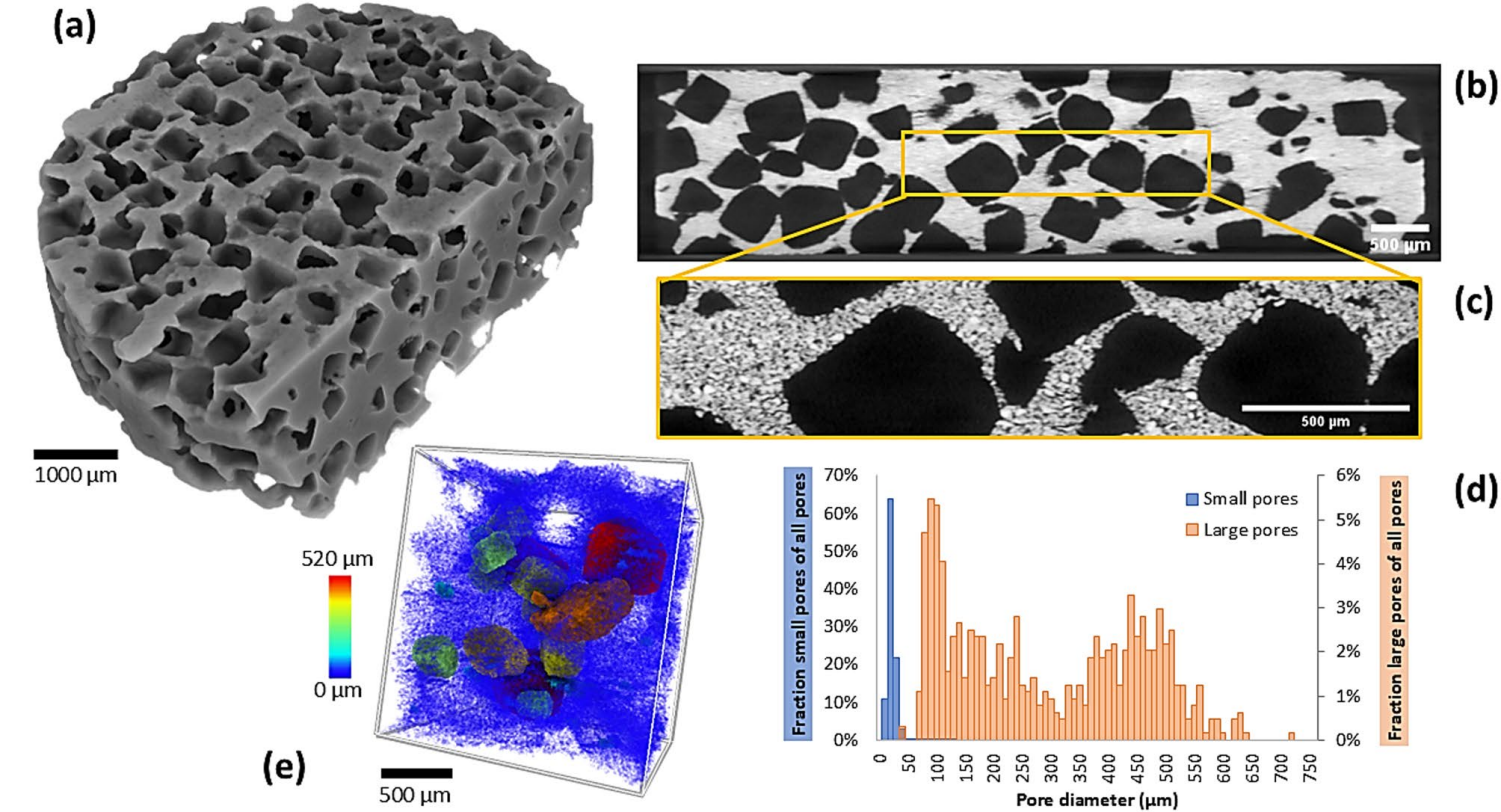
(**a**) 3D rendering from X-ray CT data of the m_Ti disc. (**b**) Cross-sectional slice image of X-ray CT data showing large cubical pores. (**c**) The expanded image of the same region shows microscale porosity in the compacted metal. (**d**) Pore size distribution histogram for both small and large pores. (**e**) Visual representation of the pores, coloured by size. Large pores touching the surface have been removed prior to measuring the pore sizes.

X-ray CT imaging enabled three-dimensional (3D) visualisation and structural analysis of the Ti foams is shown in Fig. 3a. Scanning the m_Ti disc at 8.7 µm voxel size showed a foam-like architecture composed of large pores separated by compacted material struts. The mean strut thickness was 209 ± 85 µm, and the mean pore size at this resolution was 290 ± 164 µm.

Higher-resolution scanning (voxel size 2.35 µm) revealed porosity on two length scales. While the lower-resolution scan captured only the larger pores (Fig. 3b), the higher-resolution scan showed additional micro-porosity within the compacted struts (Fig. 3c). The larger pores contributed approximately 57.6% porosity, and the microscale pores added a further 15.4%, giving an overall porosity of 73%.

Nearly all pores were interconnected, with only 0.01% fully enclosed by material. Such high interconnectivity is expected to facilitate fluid penetration throughout the scaffold, which is important for both uniform silanisation/NO charging and for nutrient and metabolite transport during biological testing. Pore size analysis showed a bimodal distribution: larger, cubical pores predominantly between 100 and 700 µm, corresponding to the NaCl particle size used during fabrication, and smaller pores ranging from a few micrometres to below 120 µm, with a mean size of 17 ± 6 µm. The pore size distributions are presented in Fig. 3d, with spatial visualisation shown in Fig. 3e. A video of the CT slice sequence is provided in the Supporting Information (Video 1).

### Surface area

**Fig. 4:**
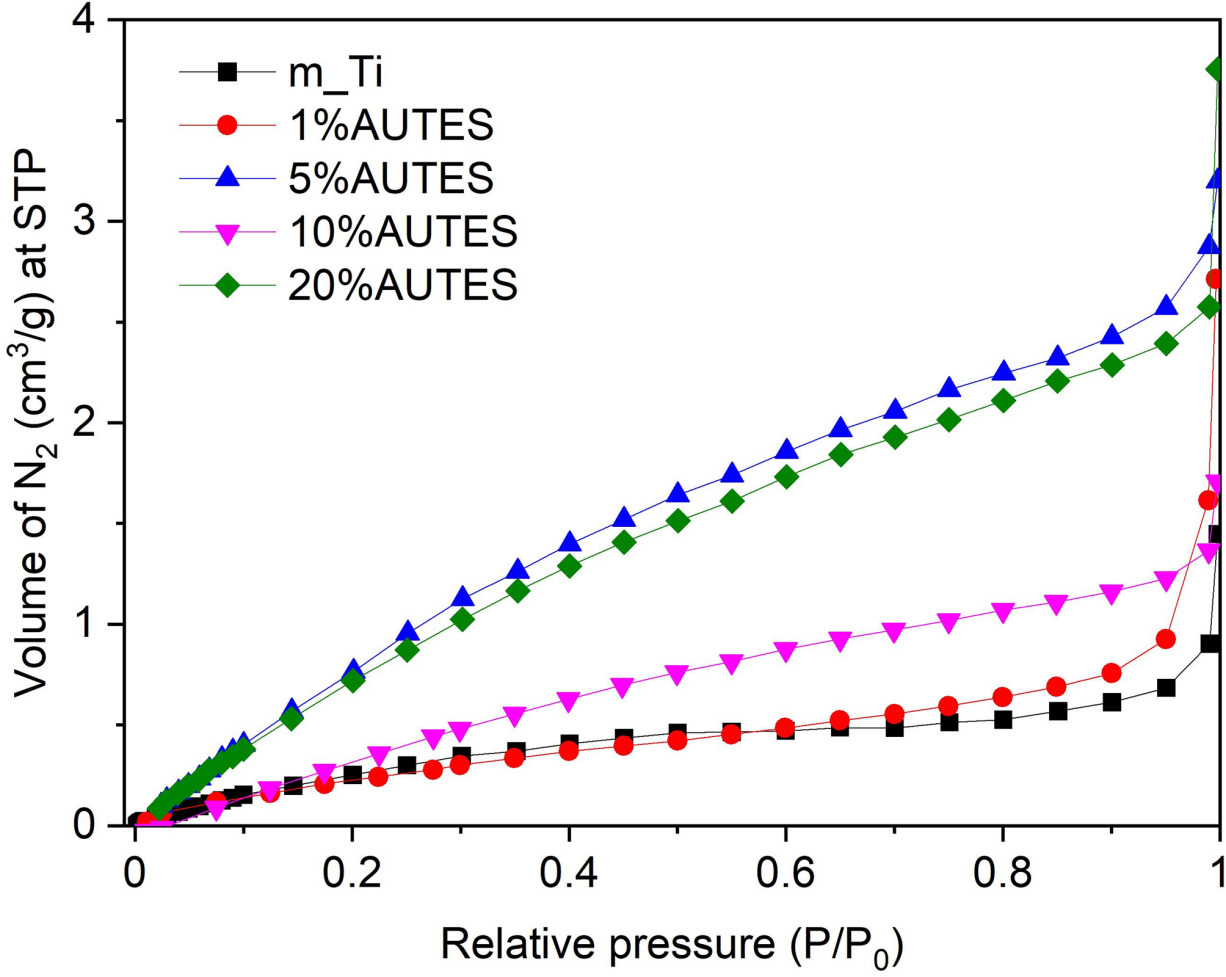
Nitrogen adsorption isothermal plots of m_Ti (black, square), 1%AUTES (red, round), 5%AUTES (blue, up-triangle), 10%AUTES (pink, down-triangle), and 20%AUTES (olive, square).

Specific surface areas of the Ti foams were quantified using BET analysis. Nitrogen adsorption isotherms of m_Ti and AUTES-functionalised foams (1–20%) are shown in Fig. 4. All samples exhibited a gradual increase in nitrogen uptake across the relative pressure range, with limited adsorption at low P/P₀ and a more pronounced rise at intermediate-to-high P/P₀. This behaviour is consistent with multilayer adsorption on rough, non-microporous surfaces rather than dominant micropore filling, which aligns with the microscale pore architecture observed by X-ray CT. The specific surface area of m_Ti was 1.5 m^2^ g^−1^. Following silanisation, surface areas increased to 6.1, 5.0, and 5.2 m^2^ g^−1^ for 5%, 10%, and 20% AUTES, respectively, indicating that surface modification increased the accessible area. The increase in BET surface area after silanisation suggests that the AUTES layer contributes additional accessible adsorption sites. This likely arises from changes in surface chemistry and topography following silane attachment, which can modify surface roughness and alter the interaction between nitrogen molecules and the foam surface.

Interestingly, the surface area did not increase proportionally with increasing AUTES concentration. The highest surface area was measured at 5% AUTES, followed by slightly lower values at 10% and 20%. This non-linear trend suggests that increasing silane concentration does not simply translate to progressively higher accessible area. At higher concentrations, changes associated with the AUTES treatment may alter nitrogen accessibility to internal features within the porous network. This interpretation is consistent with the previously observed non-linear relationship between AUTES concentration and NO payload.

For comparison, the geometric surface area of m_Ti is approximately 20 cm^2^ g^−1^ (0.002 m^2^ g^−1^)^46,47^. The substantially higher BET values therefore confirm the presence of extensive internal roughness and microscale features generated during SDP processing. These internal features significantly increase accessible surface area beyond simple geometric estimates and provide additional reactive sites for chemical functionalisation. The increased accessible area measured by BET correlates with the higher NO payloads observed in subsequent chemiluminescence studies. Together, these findings support the interpretation that the hierarchical porous architecture produced by SDP enhances functionalisation capacity and contributes directly to the observed antibiofilm performance.

### Chemiluminescence measurement and mechanism of NO release

**Fig. 5:**
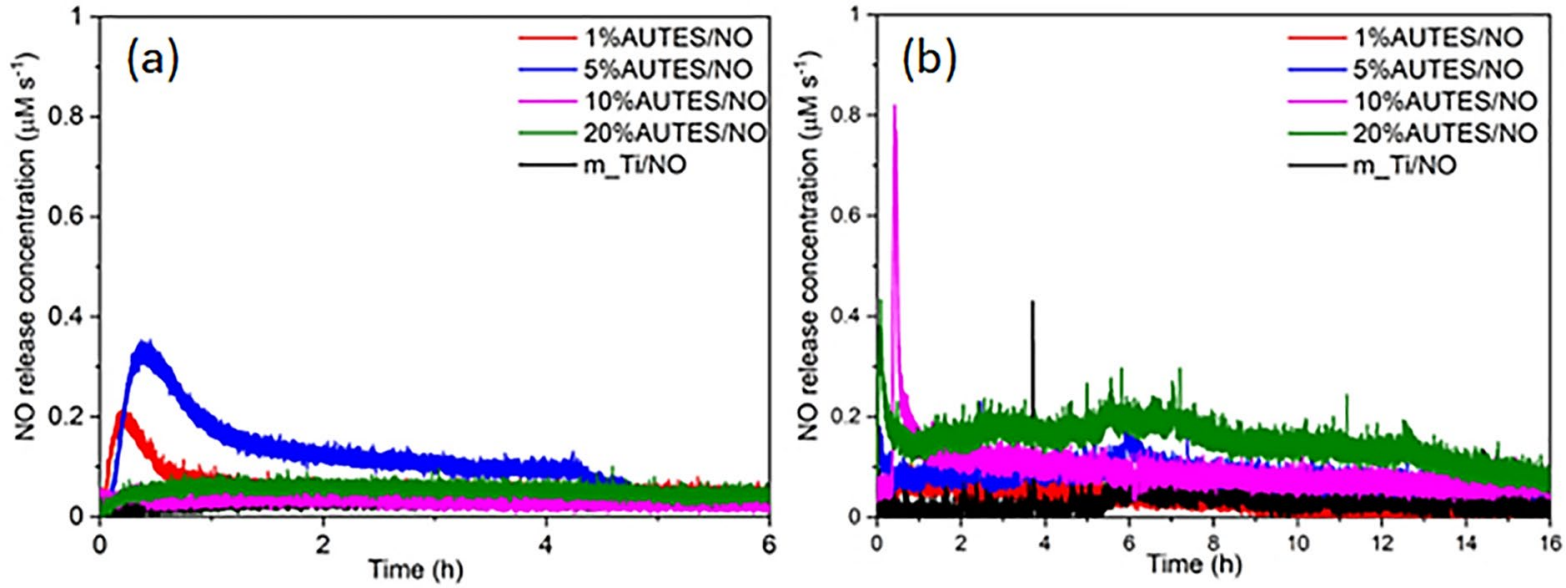
Chemiluminescent NO release profiles of *N*-diazeniumdiolate functionalised Ti foams during (**a**) 6 h in acetate buffer (pH 4) at room temperature and (**b**) 16 h in LB broth at room temperature.

The NO payload from the *N*-diazeniumdiolate functionalised Ti foams was measured using a chemiluminescence NO analyser in acetate buffer at pH 4 and LB broth medium at pH 7. These two environments were selected to separately quantify total stored NO under accelerated decomposition (pH 4) and to evaluate release behaviour under biologically relevant antimicrobial assay conditions (LB broth). The pH 4 condition was used as we have previously shown that NO is completely burst released at this pH, which enables quantification^38^. The NO releasing profiles of the Ti foams at pH 4 are shown in Fig. 5a. The kinetics of NO release, including the total concentration of NO (t[NO]), maximum instantaneous NO release concentration ([NO]_m_), time required to reach [NO]_m_ (*t*_m_), and NO release duration (*t*_d_), were determined for each modified foam, and the values are summarised in Table 1. The successful incorporation of *N*-diazeniumdiolates was confirmed functionally by chemiluminescence analysis, with negligible NO release from m_Ti controls confirming the absence of NO donor tethering.

**Table 1:** Kinetics of NO release from Ti foams in acetate buffer (pH 4) and in LB broth medium (pH 7).

Medium	Sample	t[NO]	[NO]_m_	*t*_m_	*t*_d_
		(mM)	(µM s^−1^ cm^−3^)	(min)	(h)
Acetate buffer (pH 4)	1%AUTES/NO	1.2	4.0	13	6 +
	5%AUTES/NO	2.3	6.2	22	5 +
	10%AUTES/NO	0.9	1.1	28	10 +
	20%AUTES/NO	2.0	1.5	79	14 +
	m_Ti/NO	0.4	0.9	177	4 +
LB broth (pH 7)	1%AUTES/NO	1.3	2.3	45	15 +
	5%AUTES/NO	1.9	4.1	357	15 +
	10%AUTES/NO	5.5	14.4	26	15 +
	20%AUTES/NO	8.9	7.6	4	15 +
	m_Ti/NO	0.4	1.4	363	15 +

*t[NO]: total concentration of NO (mM); [NO]_m_: maximum instantaneous NO release concentration (µM s^−1^ cm^−3^); *t*_m_: time required to reach [NO]_m_ (min); *t*_d_: NO release duration (h).

#### NO release in acetate buffer (pH 4)

NO release was first quantified in acetate buffer at pH 4 to determine the total stored NO payload of each formulation. Acidic conditions protonate the secondary amine sites of the *N*-diazeniumdiolates , rapidly destabilising them and promoting spontaneous decomposition to liberate NO. Under these conditions, NO is released in a near-complete burst, enabling accurate quantification of the total NO loading independent of biological variability.

As shown in Table 1, the maximum instantaneous NO concentration and the total NO release concentration for 1%AUTES/NO, 5%AUTES/NO, 10%AUTES/NO, 20%AUTES/NO, and m_Ti/NO were (4, 6.2, 1.1, 1.5, and 0.9 µM s^−1^ cm^−3^) and (1.2, 2.3, 0.9, 2, and 0.4 mM), respectively, in acetate buffer (pH 4). The kinetic parameters further support this behaviour. The time required to reach maximum instantaneous NO concentration (t_m_) ranged from 13 to 177 min, and the NO release duration (t_d_) ranged from 5 to 14 h. In general, both t_m_ (13–177 min) and t_d_ (5–14 h) increased with increasing AUTES concentration at pH 4, indicating progressively moderated release behaviour at higher silane loadings. As the control m_Ti group contains no amine functionality, the NO payload measured from m_Ti/NO (0.4 mM) is attributed to physisorbed NO species on the surface rather than covalently tethered *N*-diazeniumdiolate donors.

#### NO release in LB broth medium (pH 7)

NO release was also evaluated in LB broth (pH 7) to replicate the biological environment used for the antimicrobial and biofilm assays. Measuring release under these conditions provides a biologically and experimentally relevant assessment of NO flux and duration during bacterial exposure, enabling direct correlation between release kinetics and antimicrobial performance.

As shown in Table 1, the maximum instantaneous NO concentration and total NO release concentration in LB broth medium for 1%AUTES/NO, 5%AUTES/NO, 10%AUTES/NO, 20%AUTES/NO, and m_Ti/NO were (2.3, 4.1, 14.4, 7.6, and 1.4 µM s^−1^ cm^−3^) and (1.3, 1.9, 5.5, 8.9, and 0.4 mM), respectively. All formulations exhibited sustained NO release in LB broth (~ 15 h). The total NO released increased with increasing AUTES concentration, ranging from 1.3 to 8.9 mM for AUTES-functionalised samples, compared with 0.4 mM for the control m_Ti foam. These results confirm that covalent AUTES functionalisation substantially enhances NO loading and sustained release under conditions directly relevant to the bacterial experiments. The magnitude and duration of NO release observed in LB broth fall within ranges previously reported to inhibit biofilm formation, supporting the biological relevance of the measured flux and cumulative payload.

#### Mechanism of the NO release from Ti foams

The Ti foams immobilised with varying concentrations of AUTES exhibited distinct NO release profiles that reflect the interplay between porous architecture, surface functionalisation, and donor loading. At AUTES concentrations below 10%, the foams displayed a pronounced burst release of NO in acetate buffer, consistent with rapid proton-triggered decomposition of the *N*-diazeniumdiolates and efficient diffusion of NO through the highly interconnected porous network identified by X-CT. This behaviour indicates that the open pore structure permits unobstructed fluid penetration and rapid transport of released NO.

The total NO concentration released from 5%AUTES/NO was 2.3 mM, approximately twice that of 1%AUTES/NO (1.2 mM). Although the AUTES concentration increased fivefold between these samples, the NO payload did not increase proportionally. This non-linear trend suggests that increasing silane concentration does not directly translate to proportional increases in effective donor incorporation. Possible contributing factors include saturation of available Ti–OH sites, steric crowding of grafted AUTES chains, partial silane self-condensation or multilayer formation, and diffusion constraints within the porous network that reduce the fraction of amine groups converted to *N*-diazeniumdiolates .

The total NO payload increased with silane concentration up to 5% and then decreased at 10% silanisation. This reduction may reflect changes associated with higher AUTES concentrations that influence NO donor loading. In contrast, the 20%AUTES/NO foam exhibited a sustained and stable NO release for over 6 h (Fig. 5a), with a total NO release of 2 mM in acetate buffer. This behaviour suggests that at this concentration, higher donor incorporation is achieved while maintaining sufficient pore interconnectivity to allow continued NO diffusion.

Differences in release behaviour were also evident in LB broth. The 1%AUTES/NO foam reached a peak release rate of 2.3 µM s^−1^ cm^−3^ at 45 min, with a total release of 1.3 mM over 15 h. In comparison, the 20%AUTES/NO coating exhibited a higher maximum release rate of 7.6 µM s^−1^ cm^−3^ within 4 min and a substantially greater total release of 8.9 mM over 15 h. These results demonstrate that increasing AUTES concentration enhances both instantaneous NO flux and cumulative payload under biologically relevant conditions. The sharper initial release observed in LB broth may reflect improved wetting and medium penetration within the porous structure.

Overall, AUTES concentration governs both the magnitude and duration of NO delivery, with lower concentrations favouring rapid early release and higher concentrations providing increased total NO availability and prolonged release. Because NO-mediated antimicrobial activity depends on both local concentration and exposure duration at the material–bacteria interface, these distinct release profiles are expected to directly influence biofilm inhibition and bacterial viability, as examined in the following section.

### Antimicrobial analysis

The NO release profiles described above indicate that AUTES concentration modulates both instantaneous NO flux and cumulative payload under biologically relevant conditions. As antimicrobial efficacy depends on the local concentration and duration of NO exposure at the material–bacteria interface, the differing release behaviours observed in LB broth were expected to influence biofilm inhibition performance.

**Fig. 6:**
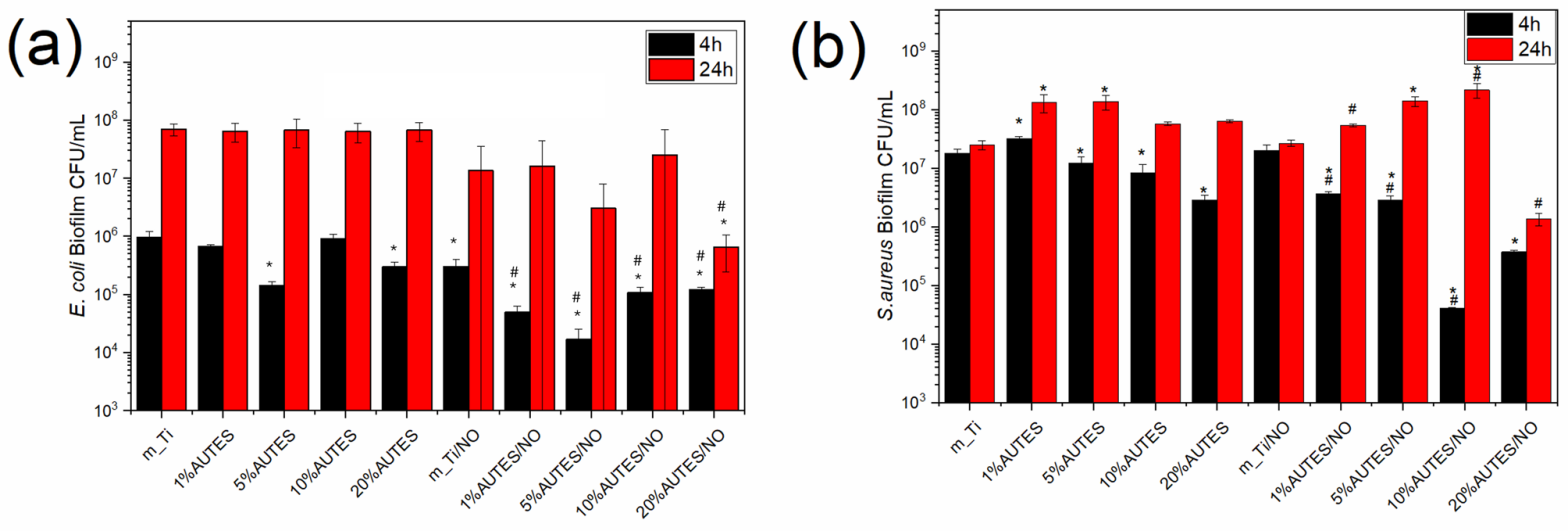
(**a**) *E. coli* and (**b**) *S. aureus* colonies formation on the NO releasing Ti foams (biofilm-associated bacteria) after 4 h and 24 h incubation. *p < 0.05 from the control m_Ti; #*p* < 0.05 from the corresponding silane concentration with no *N*-diazeniumdiolate functionalisation. (n = 3).

Approximately 80% of biomaterial-associated infections in orthopaedic surgeries are caused by bacterial biofilms^48^, which protect bacteria from immune clearance and substantially reduce susceptibility to antimicrobial agents. Preventing early surface colonisation and limiting sustained biofilm development are therefore critical for implant longevity. To evaluate whether the NO-releasing Ti foams could suppress surface-associated bacterial growth, antibiofilm activity was assessed against Gram-negative *E. coli* and Gram-positive *S. aureus*, both clinically relevant pathogens frequently implicated in orthopaedic implant infections^49,50^. A biofilm prevention assay was employed to determine whether the foams could inhibit early biofilm formation and longer-term bacterial persistence. Samples were incubated with bacterial suspensions for either 4 h or 24 h to allow biofilm development, after which planktonic bacteria were removed and the remaining biofilm-associated cells were detached and quantified using the Miles and Misra method (Fig. 6). The 4 h time point was selected because *N*-diazeniumdiolates typically exhibit burst release of a large fraction of their NO payload within this period, whereas 24 h allows sufficient time for biofilm establishment and regrowth.

For *E. coli*, the NO-releasing Ti foams demonstrated clear antibiofilm activity at both time points (Fig. 6a). After 4 h, the m_Ti/NO control produced an approximately 0.6 log reduction relative to m_Ti, likely arising primarily from small amounts of physisorbed NO. As discussed previously, the intrinsic hierarchical architecture of the SDP-derived foams may also contribute modestly by limiting stable bacterial adhesion due to surface roughness and interconnectivity. However, substantially greater reductions were observed for the AUTES/NO formulations, indicating that covalently tethered NO donors are the dominant antimicrobial mechanism.

At 4 h, 1%AUTES/NO and 5%AUTES/NO achieved approximately 1.5 and 1.8 log reductions in biofilm-associated bacteria, respectively. This enhanced early performance aligns with their more pronounced burst release behaviour^51,52^, where rapid NO flux is delivered during the initial exposure period. In contrast, 10%AUTES/NO and 20%AUTES/NO produced more modest reductions of approximately 0.8 log at this early stage, consistent with their comparatively moderated initial release profiles.

After 24 h, partial bacterial regrowth was observed across all samples, reflecting the finite NO payload and the dynamic nature of biofilm development. Nevertheless, 5%AUTES/NO and 20%AUTES/NO maintained approximately 1.5 and 2 log reductions, respectively, relative to the m_Ti control. These sustained reductions exceeded those observed for m_Ti/NO, confirming that physisorbed NO alone is insufficient for prolonged inhibition. The improved longer-term performance of 20%AUTES/NO correlates with its higher total NO release in LB broth (8.9 mM) and sustained release over ~ 15 h, indicating that extended NO availability enhances resistance to biofilm persistence.

Similar trends were observed for *S. aureus* (Fig. 6b). After 4 h, all AUTES/NO formulations inhibited biofilm-associated growth compared with both m_Ti and m_Ti/NO controls. The greatest early activity was observed for 10%AUTES/NO, which produced approximately 2.7 and 2.8 log reductions relative to m_Ti and m_Ti/NO, respectively. This strong early efficacy corresponds with its high maximum instantaneous NO release concentration of 14.4 µM s^−1^ cm^−3^, demonstrating that elevated early NO flux can produce substantial short-term biofilm suppression. As with *E. coli*, the porous architecture may assist in limiting initial adhesion; however, the magnitude of reduction observed for AUTES/NO samples indicates that NO delivery remains the principal antimicrobial factor.

Following 24 h incubation, regrowth was again detected; however, the 20%AUTES/NO formulation significantly (*P* < 0.05) inhibited regrowth compared with the corresponding silane-only control, demonstrating persistent antimicrobial activity. This behaviour is consistent with its larger cumulative NO payload and sustained release profile, which provide prolonged exposure at the material–bacteria interface.

Planktonic antimicrobial activity was also assessed. In contrast to the biofilm results, the NO-releasing foams showed limited effects against planktonic *E. coli* and *S. aureus* (Supplementary Fig. S3). Only 20%AUTES/NO produced a modest 1.1 log reduction in planktonic *E. coli* after 24 h, while reductions for planktonic *S. aureus* were ≤ 0.5 log and recovered by 24 h. This limited planktonic activity likely reflects the short half-life of NO and its restricted diffusion within the bulk culture medium, resulting in localisation of bactericidal concentrations near the foam surface^53,54^.

Together, these findings demonstrate that the NO-releasing porous Ti foams preferentially inhibit surface-associated biofilm formation rather than exert broad planktonic bactericidal effects. Importantly, the differing antibiofilm outcomes closely mirror the NO release kinetics described in “Chemiluminescence measurement and mechanism of NO release” section, confirming that AUTES concentration governs not only NO delivery profiles but also biological performance. The porous SDP architecture therefore functions as both a mechanical scaffold and a controlled chemical delivery platform, enabling localised antibiofilm activity while maintaining cytocompatibility, as discussed in the following section.

### hMSCs adhesion, morphology, and cell viability

To assess the cytocompatibility of the NO-releasing Ti foams, their ability to support viable human mesenchymal stem cells (hMSCs) was evaluated. hMSCs are multipotent progenitor cells capable of differentiating into multiple phenotypes *in vitro* and contributing to mesodermal tissues such as bone, cartilage, fat, tendons, and ligaments, as well as ectodermal tissues including nerve and liver^55^. Their response to biomaterial surfaces is therefore widely used as an indicator of osteogenic compatibility and overall implant suitability.

**Fig. 7:**
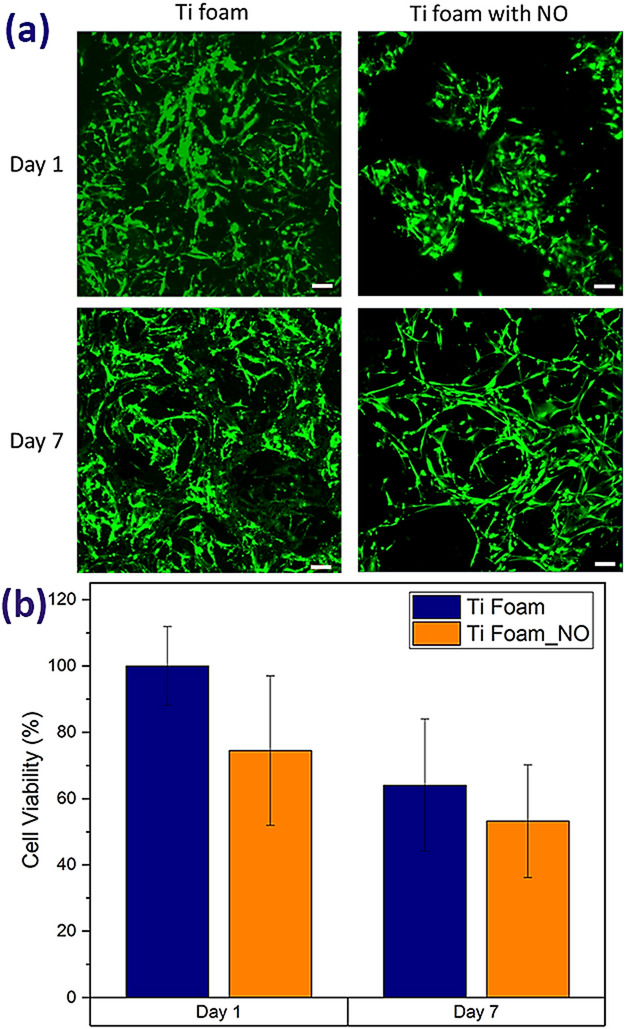
(**a**) Representative fluorescence images of the human mesenchymal stem cells (hMSCs) spreading on the m_Ti and 20%AUTES/NO foams at 1 day and 7 days post-incubation. (*n* = 3 per group). Scale bar: 100 μm, Calcein AM (green). (**b**) Cellular viability of the hMSCs in contact with the m_Ti and 20%AUTES/NO foams at day 1 and 7 as assessed by the MTT assay (n = 3, means ± SDs).

The porous Ti formulation with the highest aminosilane concentration, 20%AUTES/NO, which demonstrated the most sustained antimicrobial performance and highest cumulative NO release in LB broth, was selected to evaluate hMSC adhesion behaviour and viability. Cell attachment and morphology on the control m_Ti and 20%AUTES/NO foams were assessed over 7 days using Live/Dead staining (Fig. 7a) and the MTT metabolic activity assay (Fig. 7b).

Fluorescence imaging revealed efficient cell attachment and spreading on both substrates at day 1, with well-distributed viable cells observed across the porous surfaces. After 7 days, cells remained adherent and exhibited continued spreading, indicating stable cell–material interactions with both the untreated and AUTES/NO functionalised foams. No notable qualitative differences in cell morphology or surface treatment were observed between the two groups, demonstrating that AUTES/NO functionalisation did not adversely affect cell attachment or growth.

Quantitative MTT analysis further supported these observations. Cell viability on both m_Ti and 20%AUTES/NO foams remained comparable at day 1 and day 7, with no statistically significant differences detected between groups. These results indicate that, following NO release, even the highest AUTES concentration maintained cytocompatibility and supported metabolic activity throughout the culture period. Importantly, this demonstrates that the NO doses required to achieve antibiofilm efficacy did not induce detectable cytotoxicity under the same exposure conditions. As most NO release occurs within the first ~ 15 h, the cellular responses observed at later time points therefore reflect interactions with the functionalised surface following *N*-diazeniumdolate decomposition rather than prolonged exposure to active NO flux.

Minor reductions in metabolic activity observed at later time points are likely attributable to nutrient depletion and accumulation of waste products, as the culture medium was not refreshed during the experiment. Medium replacement was intentionally avoided to prevent altering the specific NO release behaviour of the formulations and confounding interpretation of cytocompatibility results.

Materials are generally considered cytocompatible when more than 70% of cells remain viable post-treatment^56^. Both m_Ti and 20%AUTES/NO foams exceeded this threshold throughout the study. SEM images of viable hMSCs on m_Ti and 20%AUTES/NO foams at day 7 (Supplementary Fig. S4) further confirm successful cell adhesion and spreading on the porous architecture.

## Discussion

The primary objective of this study was to determine whether SDP-derived porous titanium foams could integrate architectural control with aminosilane linkers and *N*-diazeniumdiolate donors to deliver antibacterial NO while maintaining initial cytocompatibility. The sintering–dissolution process generated a highly interconnected, hierarchically porous architecture with substantially greater accessible surface area. This amplified internal surface area enhanced *N*-diazeniumdiolate donor loading, resulting in tunable NO release profiles.

Increasing AUTES concentration modulated both instantaneous NO flux and cumulative payload in biologically relevant media, directly influencing antibiofilm performance. Because NO release was measured in media that differs chemically from those used in the biological assays, direct comparison between release kinetics and cellular or antibacterial responses is limited. The release profiles therefore provide a useful indication of relative behaviour across formulations, but not a direct quantitative match to the biological environments. Formulations exhibiting sustained NO release maintained significant inhibition of biofilm-associated *E. coli* and *S. aureus* while preserving hMSC viability over 7 days. Together, these findings demonstrate that the porous SDP architecture functions as a platform for chemical modification and controlled antimicrobial delivery without compromising cytocompatibility.

These results align with the broader rationale for porous titanium implants, where reducing stiffness mismatch and increasing surface area promote mechanical compatibility and biological interlocking with surrounding bone. In addition to architectural optimisation, multifunctional surface chemistries are increasingly required to address implant-associated infection while supporting tissue integration. Previous work from our group demonstrated that the NO donor *N*-diazeniumdiolate can be tethered onto solid Ti via aminosilane linkers, providing effective antimicrobial activity against *S. aureus* and *P. aeruginosa* while supporting human foetal osteoblast growth following NO release^34^. Curran et al. have demonstrated that long-chain aminosilanes bearing pendant amine groups promote formation of an apatite-like layer that modulates cell responses and induces osteogenic differentiation of mesenchymal stem cells, resulting in high-quality de novo tissue formation^38,39^. Building directly on this foundation, the present study applies the sintering–dissolution process (SDP) as a scalable manufacturing route to produce highly interconnected porous foams that not only satisfy structural requirements for bone ingrowth but also provide an expanded surface for effective chemical functionalisation and controlled localised antimicrobial delivery.

Here, the sintering-dissolution process (SDP) was applied for the first time to fabricate biomedical porous Ti foams and exploited to enhance chemical functionalisation capacity and antimicrobial performance. Unlike additive manufacturing approaches that rely on specialised equipment and high energy input, SDP provides a simple and economical powder metallurgy route capable of producing highly interconnected porous structures with controllable pore size, shape, and interconnectivity. Although mechanical properties were not directly measured in this study, prior work on SDP-derived metallic foams demonstrates tunable modulus and compressive strength within ranges suitable for load-bearing applications^21,22^, supporting the translational relevance of this architecture.

From a design perspective, porous Ti foams must possess pore sizes that support tissue ingrowth, oxygen and nutrient transport, and space for cell migration, proliferation, and differentiation^57^. Effective designs also require sufficient porosity to enable vascularisation while minimising material usage and maintaining a biomimetic profile comparable to bone (50–90% porosity)^58,59^. In general, higher porosity (> 70%) enhances bone ingrowth compared with lower porosity (< 70%)^58^. For example, Markoff et al. reported that 75% porosity with an open structure maximised osteoblast activity and migration, while Di Luca et al. showed that hMSCs cultured on porous gradient scaffolds exhibited increased osteogenic differentiation at larger pore sizes due to improved nutrient and oxygen transport^60^.

The SDP-manufactured foams in this study exhibited a bimodal pore distribution, with 57.6% porosity from larger pores and 15.4% from smaller pores, resulting in an overall porosity of 73%. Nearly all pores were interconnected, with only 0.01% fully enclosed. Such high interconnectivity is expected to facilitate fluid penetration throughout the scaffold, which is important for both uniform silanisation/NO tethering and for nutrient and metabolite transport during biological testing. Larger pores ranged from 300 to 700 µm, while smaller pores were below 300 µm. These dimensions fall within the range considered favourable for osteoblast adhesion, migration, and proliferation (200–400 µm), with pores larger than 300 µm known to enhance new bone and capillary formation^61^. The interconnected architecture further supports body fluid transport and vascularisation. Consistent with these structural characteristics, hMSC culture on the SDP foams demonstrated cytocompatibility over 7 days, confirming that the architecture is compatible with cell survival and attachment.

Although porous implants have been widely investigated, relatively few studies have combined enhanced osseointegration with antimicrobial functionality. Badar et al. reported porous Ti alloy discs coated with ciprofloxacin-containing double hydroxide layers that exhibited antimicrobial efficacy against *P. aeruginosa* together with in vitro cytocompatibility^62^. Yaravi et al. developed porous Ti scaffolds eluting silver ions that eradicated *S. aureus* and inhibited biofilm formation; however, burst release of silver resulted in cytotoxicity during the first day of culture^63^. These examples highlight the difficulty of achieving sustained antimicrobial efficacy without compromising cell compatibility.

In contrast, the present study demonstrates that AUTES-functionalised porous Ti foams can be successfully coupled with NO-releasing *N*-diazeniumdiolates to provide antibiofilm activity while maintaining cytocompatibility. The NO-releasing foams significantly reduced biofilm-associated bacteria, achieving up to 2.8 and 2 log reductions in biofilm-adhered *S. aureus* and *E. coli*, respectively. Differences between formulations were closely correlated with their NO release kinetics. At lower silane loading, 5%AUTES/NO exhibited a more pronounced early burst release in LB broth, reaching a maximum instantaneous flux of 4.1 µM s^−1^ cm^−3^ and delivering a total payload of 1.9 mM, which corresponded to stronger early antibiofilm reductions at 4 h. In contrast, the 20%AUTES/NO foams generated a substantially higher total NO payload of 8.9 mM with sustained release over approximately 15 h, despite a peak flux of 7 µM s^−1^ cm^−3^ occurring within the first few minutes. This behaviour is consistent with higher AUTES concentrations being associated with increased NO donor loading and extended release duration, although the underlying surface‑level changes cannot be confirmed from the present data. Consequently, although intermediate formulations promoted rapid early reductions, the 20%AUTES/NO foams maintained significant inhibition after 24 h (*P* < 0.05), indicating more persistent protection against biofilm establishment. As implant-associated infections are primarily driven by sustained surface colonisation rather than short-term bacterial exposure, prolonged localised NO delivery is likely to be more clinically relevant than an initial burst, supporting selection of the 20%AUTES/NO formulation as the optimal configuration. Importantly, even at this highest silane concentration, the foams continued to support viable hMSC adhesion over 7 days, demonstrating that enhanced antimicrobial performance did not compromise cytocompatibility.

Notably, the antimicrobial performance of the porous Ti foams exceeded that previously observed for solid Ti surfaces. Our earlier work on AUTES-coated solid Ti showed only a 0.8 log reduction for *S. aureus* and no antibiofilm efficacy against *P. aeruginosa*^34^. In comparison, the porous foams in this study achieved substantially greater reductions. This improvement is likely attributable to the increased internal surface area of the SDP foams, which enable higher donor loading and more efficient localised NO delivery. These findings therefore demonstrate a structure-enabled relationship between processing, structure, and function, whereby the SDP-derived architecture enhances both chemical functionalisation and biological performance.

Overall, the results show that SDP-fabricated porous Ti foams can act as effective carriers for tethered NO donors, enabling localised antibiofilm activity while maintaining initial mammalian cell compatibility. The study demonstrates the feasibility of combining a scalable fabrication method with chemical surface modification to achieve multifunctionality within a single platform.

## Conclusions

The development of porous titanium implants that resist infection while remaining compatible with host tissue remains a significant challenge in orthopaedic biomaterials. In this study, a cost-effective sintering-dissolution process (SDP) was successfully applied to a biomedical context to fabricate highly interconnected porous Ti foams with approximately 73% total porosity and near-complete interconnectivity. The resulting hierarchical architecture provided high surface area, and pore dimensions suitable for fluid transport and cell attachment, while maintaining cytocompatibility with human mesenchymal stem cells over 7 days.

Surface functionalisation with the aminosilane AUTES and tethered NO-releasing *N*-diazeniumdiolates enabled localised antimicrobial delivery without compromising cell viability. The functionalised foams exhibited high initial NO flux and sustained release for more than 15 h, producing significant reductions in biofilm-adhered *E. coli* and *S. aureus* while showing limited activity against planktonic bacteria. Among the formulations tested, the 20% AUTES/NO foams provided the most sustained NO release and maintained significant antibiofilm efficacy after 24 h (*p* < 0.05), while continuing to support stem cell adhesion and survival. This performance arises from the higher donor loading and prolonged release kinetics enabled by the increased functionalised surface area of the porous architecture.

Overall these results demonstrate that SDP-derived porous Ti foams can integrate structural compliance, selective antibiofilm functionality and initial cytocompatibility within a single platform. The successful coupling of scalable manufacturing with multifunctional surface engineering demonstrates that this approach provides a promising practical and clinically relevant pathway towards infection-resistant orthopaedic implants.

## Supplementary Information

Below is the link to the electronic supplementary material.

Supplementary Material 1

Supplementary Material 2

## Data Availability

The datasets used and/or analysed during the current study are available from the first or corresponding author on reasonable request.
