# Supplementary material for: Nitric oxide‑releasing porous titanium foams fabricated by scalable sintering–dissolution process for antibiofilm activity and cytocompatibility
Source: Sci Rep. 2026 Apr 22;16:18636. doi: 10.1038/s41598-026-47080-x (PMC13269483; doi:10.1038/s41598-026-47080-x)
Supplement: Supplementary file 2 — Supplementary Material 2 [file 41598_2026_47080_MOESM2_ESM.docx]

Nitric oxide‑releasing porous titanium foams fabricated by scalable sintering–dissolution process for antibiofilm activity and cytocompatibility

Man Li ^1,§^, Pengcheng Zhu ^1,§^, Vahid Heravi Shargh^1^, Jenny Aveyard ^1^, Manohar Koduri ^1^, Mark Hunter ^1^, Julia G. Behnsen ^1^, Yuyuan Zhao ^2^, Judith M. Curran ^1^ and Raechelle A. D’Sa ^1,^*

^1^ Department of Materials, Design and Manufacturing Engineering, University of Liverpool, Liverpool L69 3GH, UK

^2^ School of Mechanical and Automotive Engineering, Ningbo University of Technology,
Ningbo 315211, China

***** Correspondence: r.dsa@liverpool.ac.uk

§ Man Li and Pengcheng Zhu are joint first authors.

**Supplementary Information**


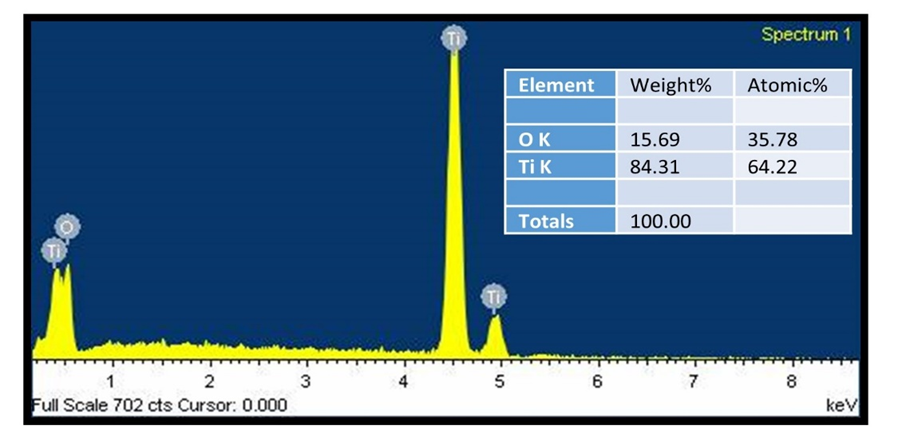


**Supplementary Figure S1.** Elemental spectrum of m_Ti. The inserted table displays the element weight percentage and atomic percentage analysis of m_Ti.


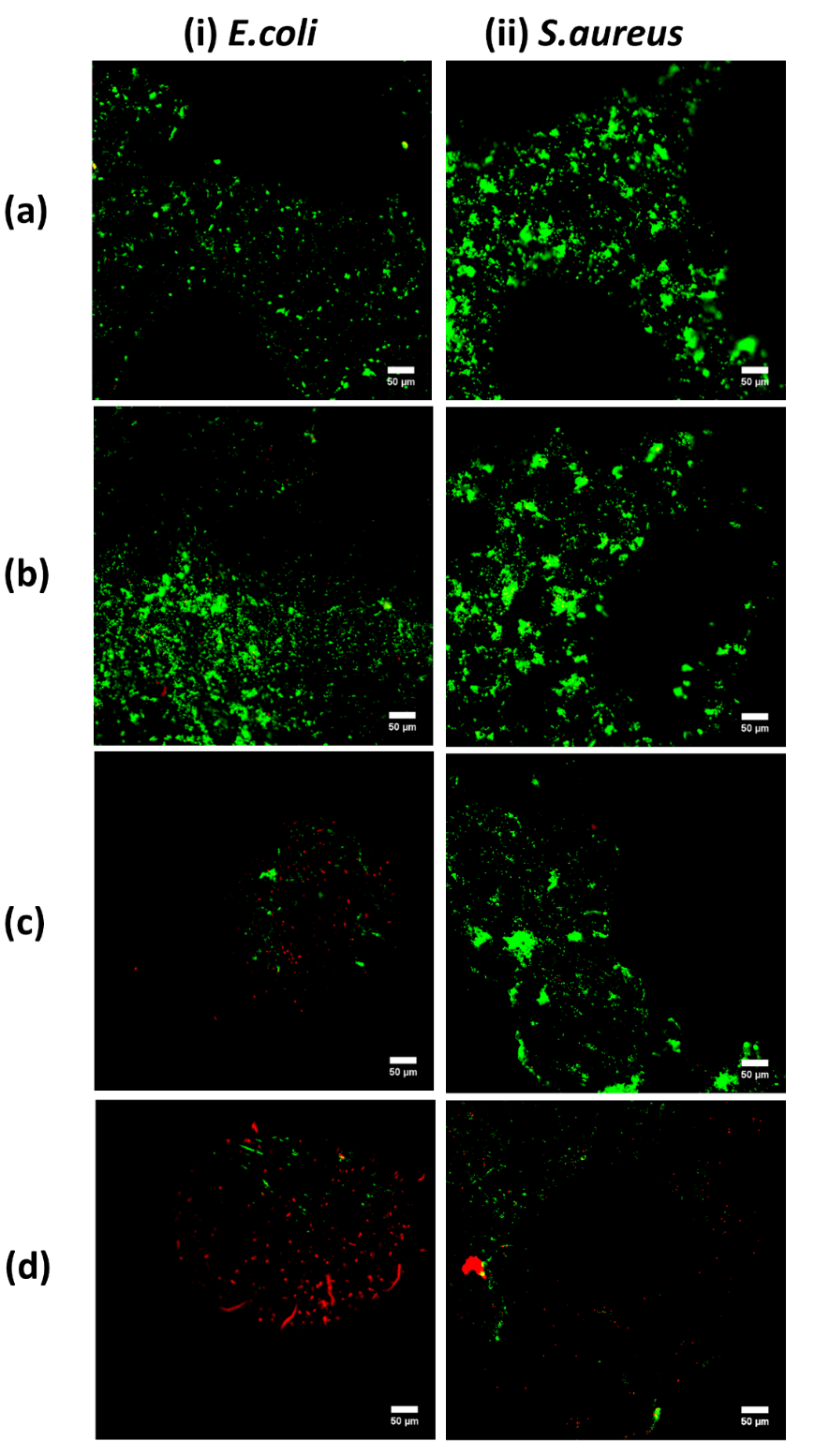


**Supplementary Figure S2.** Fluorescence images of the live/dead i) E. coli and ii) S. aureus on a) m_Ti, b) m_Ti/NO, c) 1%AUTES/NO and d) 5%AUTES/NO after 4 h incubation. Representative images are shown, n = 3 random spots per sample (scale bar: 50 µm, live bacteria: green, dead bacteria: red).





**Supplementary Figure S3.** Planktonic a) *E. coli* and b) *S. aureus* colonies formation in the medium containing the NO release Ti foams after 4 and 24 h incubation.


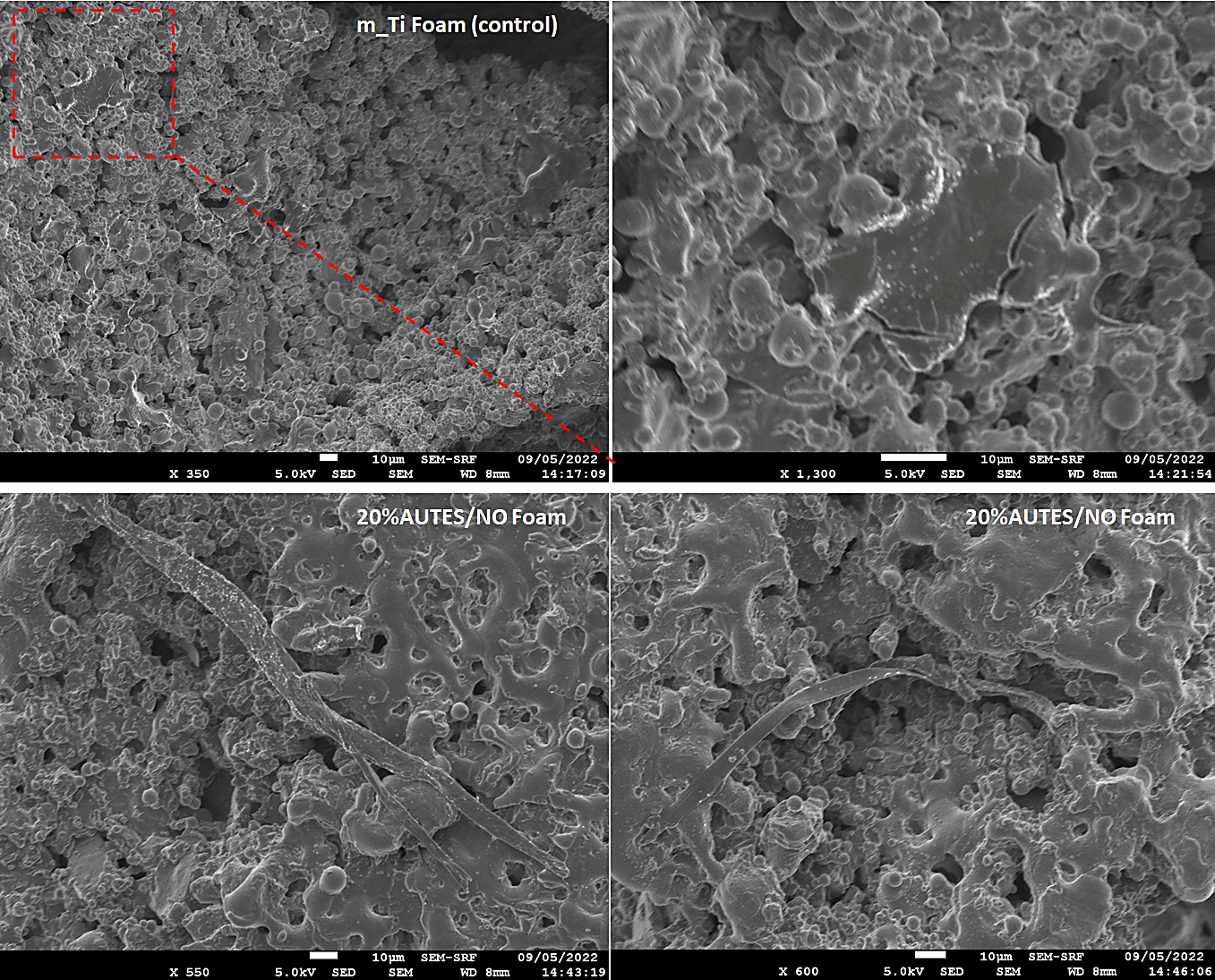


**Supplementary Figure S4.** SEM images of the viable hMSCs on the control m_Ti and the 20%AUTES/NO foams at day 7 post-incubation.
